# Autoimmune Polyendocrine Syndromes: Recognizing the Overlooked Clusters of Endocrinopathies

**DOI:** 10.7759/cureus.89936

**Published:** 2025-08-12

**Authors:** Gizem Reyhanoglu, Divya Madhavarapu, Antoni Kafrouni

**Affiliations:** 1 Internal Medicine, Tallahassee Memorial HealthCare, Tallahassee, USA; 2 Endocrinology, Albany Medical Center, New York, USA; 3 Endocrinology, Mayo Clinic, Jacksonville, USA

**Keywords:** aps type i, aps type ii, diabetes mellitus thyroid pathologies nutritional and metabolic pathologies autoimmune diseases, multiple autoimmune syndrome, thyroid disorder

## Abstract

The first case describes a patient who underwent genetic testing for autoimmune polyendocrine syndrome (APS), revealing a change in the autoimmune regulator (AIRE) gene, c.1066C>T. To diagnose classical APS-1, two of the following endocrinopathies are required: hypoparathyroidism, adrenal insufficiency, and mucocutaneous candidiasis. Due to the patient’s genetic mutation and high susceptibility to developing other endocrinopathies later in life, he is presumed to have a nonclassical APS-1 presentation given his genetic testing.

The second case discusses APS-2. This patient has a history of autoimmune diabetes, primary hypogonadism, and hypothyroidism. APS-2 is diagnosed when a patient has two of the following endocrinopathies: adrenal insufficiency, autoimmune thyroid disease, and type 1 diabetes. This patient has a strong family history of autoimmune endocrinopathies. With more knowledge and recognition of APS, complications and detrimental outcomes can be more easily predicted and prevented.

## Introduction

Autoimmune polyendocrine syndrome (APS), also known as autoimmune polyglandular syndrome, comprises rare diseases characterized by autoimmune-mediated destruction of multiple endocrine organs [1,2]. This group of syndromes is hypothesized to arise secondary to immunologically mediated destructive processes that are still not well understood [2].

The concept of autoimmune endocrinopathies co-occurring within the same individual has a long clinical history. One of the earliest documented associations was reported by Ogle in 1866, who noted a case of diabetes mellitus with adrenal insufficiency, though the adrenal pathology at the time was attributed to tuberculosis rather than autoimmunity [3,4]. Additional correlations were documented over the following decades; in 1910, Parkinson described the co-occurrence of diabetes mellitus and pernicious anemia [4,5]. In 1926, Schmidt published his influential report detailing the coexistence of hypothyroidism and Addison’s disease, both featuring lymphocytic infiltration on histology, leading to the later eponym "Schmidt’s syndrome" [4,6]. Building on these observations, Rowntree and Snell compiled multiple cases linking Addison’s disease with other autoimmune endocrine conditions, such as Hashimoto’s thyroiditis and type 1 diabetes [3,4]. The modern classification of autoimmune polyendocrine syndromes was established in 1980 when Neufeld, Maclaren, and Blizzard proposed a system that unified these clinical patterns under the umbrella term APS, categorizing them based on onset, inheritance, and associated manifestations [7].

Among the recognized forms, APS-1 and APS-2 are the most encountered subtypes [6,8]. Improved awareness among clinicians has contributed to earlier recognition and diagnosis. These syndromes may present at any age, from early childhood into late adulthood, and new autoimmune features can emerge over time. Diagnosis typically involves biochemical confirmation of glandular dysfunction, followed by screening for autoantibodies. Management is individualized and disease-specific, requiring prompt intervention to prevent life-threatening complications. In this report, we present two cases of APS, illustrating the spectrum of clinical presentations and the diagnostic challenges they pose.

## Case presentation

Written informed consent was obtained from both patients for publication of this case report and accompanying images.

Case 1

A male in his mid-40s presented with a long-standing history of autoimmune endocrinopathies, initially raising suspicion for autoimmune polyendocrine syndrome. His first endocrine manifestation occurred in his early 20s when he experienced dizziness and syncope, leading to a work-up and subsequent diagnosis of Addison's disease, confirmed by low cortisol levels and positive 21-alpha hydroxylase antibodies. He was initiated on hydrocortisone and fludrocortisone and has remained on long-term steroid therapy.

Around the same period, at age 22, he was also diagnosed with primary hypothyroidism and started on levothyroxine 150 mcg daily. A few years later, celiac disease was identified based on gastrointestinal symptoms and positive tissue transglutaminase (tTG) IgG antibodies. 

Given the clustering of autoimmune conditions, APS was suspected. Collectively, his autoimmune screenings revealed positive 21-hydroxylase antibodies (consistent with adrenal insufficiency), positive tTG antibodies (confirming celiac disease), and negative GAD-65, ZNT8, islet cell antibodies, thyroid peroxidase (TPO), and parietal cell antibodies, reducing suspicion for autoimmune diabetes or pernicious anemia. Screenings for other possible endocrine involvement showed normal parathyroid hormone (PTH) and calcium levels (no evidence of hypoparathyroidism), and normal luteinizing hormone (LH), follicle-stimulating hormone (FSH), and testosterone, suggesting no current hypogonadism. 

At the time of his initial endocrinology investigation, there was no history of mucocutaneous candidiasis, a hallmark feature of APS-1, and AIRE gene testing was negative, making classical APS-1 less likely. However, the combination of multiple autoimmune endocrinopathies without features typical of APS-2 (such as type 1 diabetes) raises the possibility of a non-classical APS-1 variant or other polygenic autoimmune syndrome. He remains under routine surveillance for evolving endocrinopathies.

Case 2

A male in his late 30s presented with multiple autoimmune conditions developing progressively over more than a decade. He was first diagnosed with vitiligo in his early 20s. In his early 30s, hypogonadism was identified, and he was started on testosterone replacement therapy. Subsequently, he was diagnosed with primary hypothyroidism (managed with levothyroxine 100 mcg daily), celiac disease (positive tTG IgG antibodies), and vitamin B12 deficiency. 

He was then seen by an endocrinologist who noted the accumulation of autoimmune features suggestive of APS. Because of this, the patient underwent further testing. Autoantibody testing revealed positive GAD-65 antibodies, raising concern for autoimmune diabetes (though he remains in a prediabetic state). Given his age of onset, presence of GAD-65 autoantibodies, and the absence of insulin requirements during the first six months after diagnosis, latent autoimmune diabetes of adults is a potential diagnosis. He also has negative 21-hydroxylase antibodies, negative islet cell antibodies, and negative ZNT8 antibodies. Other relevant labs include normal PTH, calcium, and no history of adrenal insufficiency. 

Although Addison’s disease is not currently present, the combination of autoimmune thyroid disease, hypogonadism, celiac disease, and positive GAD antibodies supports a diagnosis of probable APS-2. He is under ongoing monitoring for progression to overt type 1 diabetes or adrenal insufficiency.

The classical features of APS-1 and APS-2 are illustrated in Table 1, which provides a visual comparison to the presentations described in the above cases [1,2]. A summary of key clinical findings for both patients is presented in Tables 2, 3, allowing for side-by-side comparison of the overlapping and distinct features of each case.

**Table 1:** Comparison of the classical presentation of APS-1 and APS-2 AIRE: autoimmune regulator, HLA: human leukocyte antigen, MHC: major histocompatibility complex, APS: autoimmune polyendocrine syndrome. Citations: [1,2]

	APS-1	APS-2
Onset	Infancy	Older
Inheritance	Autosomal recessive	Polygenic
Gene mutation	AIRE gene mutation on chromosome 21	No gene mutation
HLA association	No HLA association	MHC class II: DR3, DR4
Main endocrinopathies	Addison’s disease	Addison’s disease
	Hypoparathyroidism	Autoimmune thyroid disease
	Mucocutaneous candidiasis	Type I diabetes
Frequency of diabetes	~20%	~50%
Male: Female	Equal	Females > Male

**Table 2:** Comparison of the two cases *Patient would be classified more specifically as latent autoimmune diabetes in adults (LADA), also known as type 1.5 diabetes, given the GAD antibodies and the late onset of his diagnosis, around his 30s.

	Case 1	Case 2
Addison’s disease	Present	Not present
Hypothyroidism	Present	Present
Autoimmune diabetes	Not present	Present*
Hypogonadism	Not present	Present
Celiac disease	Present	Present
Hyperparathyroidism	Not present	Not present
Chronic mucocutaneous candidiasis	Not present	Not present
Vitiligo	Not present	Present
Vitamin B12 deficiency	Not present	Present

**Table 3:** Comparison of features between cases 1 and 2 AIRE: Autoimmune regulator, NLRP: NLR family pyrin domain containing, GAD: Generalized anxiety disorder

Feature	Case 1	Case 2
Anti-21-hydroxylase Ab	Positive	Negative
Anti-thyroid peroxidase	Positive	Positive
Anti-TTG IgA	Negative	Positive
GAD antibodies	Negative	Positive
Anti-parietal cell Ab	Positive	Negative
Genetic Testing Method	Next-generation sequencing (NGS) panel	Next-generation sequencing (NGS) panel
AIRE mutation	c.1006C>T (p.Arg336Cys), heterozygous	None detected
NLRP1 variant	Not detected	Variant present
Variant classification	AIRE: VUS	NLRP1: Associated with APS-2 risk

## Discussion

APS-1 and APS-2 are often underdiagnosed due to their rarity and varied presentations [6,9]. APS-1 is rarer than APS-2, affecting one in every two to three million newborns with no discernible gender predominance [10]. Increased physician awareness can lead to more valuable information about these syndromes. Although multiple APS subtypes exist, this paper focuses primarily on APS-1 and APS-2. A generalized comparison of these subtypes is presented in Table 1, and a specific comparison of the two cases discussed in this report is shown in Table 2.

APS-1, also known as autoimmune polyendocrinopathy-candidiasis-ectodermal dystrophy (APECED), is an autosomal recessive disorder caused by a mutation of the autoimmune regulator (AIRE) gene, located on the short arm of chromosome 21 [2,9,11]. The first endocrinopathy often appears in infancy, with an APS-1 diagnosis confirmed when multiple endocrine disorders develop [2,11].

The AIRE gene encodes a protein primarily involved with immune-related tissues and organs, particularly in the medullary thymic epithelial cells [10]. Mutations in AIRE result in impaired peripheral antigen expression in the thymus, reducing the deletion of autoreactive T-lymphocytes that would otherwise target peripheral antigens [8]. Notably, even siblings with the same mutation may exhibit highly variable clinical presentations [9].

The primary autoimmune manifestations of APS-1 include chronic mucocutaneous candidiasis (CMC), hypoparathyroidism, and adrenal insufficiency [10]. A diagnosis of "classical" APS-1 requires the presence of at least two of these conditions [2,10,11]. CMC is the most common initial feature, followed by hypoparathyroidism, which often presents earlier in life [9]. Symptoms indicative of hypoparathyroidism include perioral and fingertip numbness or tingling, muscle cramps, seizures, and positive Chvostek and Trousseau signs [12]. Additional autoimmune conditions associated with APS-1 include type 1 diabetes, hypergonadotropic hypogonadism, ovarian failure, and hepatitis [1]. In women with APS-1, primary ovarian insufficiency affects approximately 60% before the age of 30 [1]. Because APS-1 manifestations can emerge over time, routine surveillance for additional endocrine disorders is strongly recommended [8].

Autoantibodies against type 1 interferons, particularly interferon omega and alpha subtypes, are found in nearly all APS-1 patients, making them a highly specific screening marker [1,2]. Other disease-associated autoantibodies include glutamic acid decarboxylase-65 (GAD-65) in type 1 diabetes, 21-hydroxylase in Addison’s disease, and side-chain cleavage enzyme in autoimmune premature ovarian insufficiency [1]. Recent studies also suggest a link between parathyroid-specific NALP5 protein autoantibodies and hypoparathyroidism in APS-1 [2].

Interestingly, some APS-1 patients develop anti-GAD-65 autoantibodies without showing signs of autoimmune diabetes, likely due to GAD expression in both pancreatic islet cells and the nervous system [2]. As a result, GAD-65 may not be a reliable marker for diagnosing type 1 diabetes in APS-1 [2]. Instead, anti-insulin or anti-islet cell antigen 2 (IA2) antibodies may provide more specificity for screening [2].

Case 1 presented with Addison’s disease and primary hypothyroidism, meeting clinical criteria for autoimmune polyendocrine syndrome type 2 (APS-2). However, due to the early age of onset and identification of a heterozygous missense mutation in the AIRE gene after undergoing genetic testing (c.1006C>T), there is a possibility of a nonclassical APS-1 phenotype. While this mutation is currently classified as a variant of uncertain significance (VUS), it has been previously associated with incomplete APS-1, including cases that do not meet the classic triad. This case emphasizes the importance of genetic testing and longitudinal follow-up in diagnosing atypical APS presentations. The diagnosis of APS-2 remains likely, but the identification of an AIRE mutation raises the possibility of evolving APS-1, especially in the context of early presentation and the potential for additional autoimmune features to emerge over time.

Given the genetic nature of APS-1, it is important to screen all siblings, even those who appear healthy, for AIRE mutations and associated autoantibodies. Treatment is tailored to the specific autoimmune disorders present, but the wide variability in disease manifestations and severity makes both diagnosis and management challenging.

APS-2 is associated with class II (human leukocyte antigen) HLA alleles, particularly DR3 and DR4 [8]. Unlike APS-1, it is not caused by a single gene mutation but instead follows a complex inheritance pattern [2]. APS-2 is three times more common in women than men and tends to develop later in life compared to APS-1 [2,6]. This syndrome encompasses a broad spectrum of autoimmune disorders, including Addison’s disease, Graves’ disease, primary hypothyroidism, primary hypogonadism, hypopituitarism, IgA deficiency, autoimmune diabetes mellitus, Parkinson’s disease, celiac disease, vitiligo, pernicious anemia, and autoimmune thyroiditis [8]. A diagnosis of APS-2 is typically made when a patient presents with at least two of the following: type 1 diabetes mellitus, Addison’s disease, or autoimmune thyroid disease [2,6]. A specific subset of APS-2, known as Carpenter syndrome, is characterized by the simultaneous presence of all three endocrinopathies [6]. Among these conditions, autoimmune thyroid disease is the most frequently observed [6].

Given the complexity of APS-2 in both presentation and inheritance, identifying clear patterns among its associated disorders remains a challenge. However, type 1 diabetes, autoimmune thyroiditis, and Addison’s disease have all been linked to genetic risk factors involving the HLA, CTLA4, and PTPN22 genes [2]. The clinical presentation of APS-2 varies widely, and symptoms can be nonspecific, including weight loss, fatigue, nausea, vomiting, generalized weakness, hyperpigmentation, hypoglycemia, and orthostatic hypotension. These vague symptoms often delay diagnosis [6,8]. Diagnostic testing for APS-2 should be guided by clinical suspicion. Thyroid function can be assessed with thyroid-stimulating hormone (TSH) and free T4 levels, while primary adrenal insufficiency is best evaluated with a morning serum cortisol test or an adrenocorticotropic hormone (ACTH) stimulation test [6]. Autoantibody screening is useful in suspected cases, with common markers including thyroid peroxidase antibodies for autoimmune thyroid disease, anti-IA2 and anti-insulin antibodies for type 1 diabetes, and 21-hydroxylase antibodies for autoimmune Addison’s disease [1]. Given the hereditary component of APS-2, regular monitoring of family members is recommended to detect organ-specific dysfunction early [6].

Case 2 presented with autoimmune thyroiditis, primary hypogonadism, positive GAD antibodies, and biopsy-confirmed celiac disease. He has not yet developed adrenal insufficiency but is under surveillance for progression. Although no AIRE mutation was detected, a variant in the NLRP1 gene, with polygenic autoimmune risk and previously linked to APS-2, was identified when he underwent genetic screening. His family history includes type 1 diabetes in a sibling. The clustering of autoimmune features supports a probable diagnosis of APS-2.

Advancements in APS research continue to expand, improving diagnostic capabilities and surveillance strategies. As understanding of the disease progresses, more targeted treatments may emerge. Early recognition of endocrinopathy patterns is critical for timely intervention and prevention of life-threatening complications. Currently, clinical trials are evaluating immune modulation therapies using anti-CD3 and anti-CD20 antibodies in newly diagnosed type 1 diabetics to influence T and B cell function, though these treatments have not yet demonstrated a significant impact on disease progression [12]. Management of APS-2 primarily focuses on treating the individual autoimmune conditions as they arise. While genetic counseling is especially relevant for APS-1 due to its autosomal recessive inheritance, it may also be beneficial for families affected by APS-2 to better understand their risk of disease transmission and development [9].

## Conclusions

Following biochemical workup, both patients underwent genetic testing. Case 1 meets traditional criteria for APS-2 but also harbors a heterozygous AIRE gene variant (c.1006C>T) previously associated with nonclassical APS-1, raising the possibility of an evolving APS-1 phenotype. Routine monitoring is planned to detect additional endocrinopathies, reflecting the variable onset of APS-1 manifestations.

Case 2 demonstrates a clustering of autoimmune conditions consistent with probable APS-2, supported by the presence of an NLRP1 variant and a family history of autoimmunity. These cases highlight the clinical utility of combining classical diagnostic criteria with genetic testing and long-term follow-up, particularly in atypical presentations. Further genetic testing in his family revealed a shared NLRP1 variant among him, his brother, and his mother. The NLRP1 gene is implicated in rheumatoid arthritis, Celiac disease, Addison’s disease, and type 1 diabetes, linking it to inflammation, autoimmune disorders, and caspase-mediated apoptosis. The two patients involved in these cases are being closely monitored for the progression of other endocrinopathies. 
